# NCI’s Proteomic Data Commons: A Cloud-Based Proteomics Repository Empowering Comprehensive Cancer Analysis through Cross-Referencing with Genomic and Imaging Data

**DOI:** 10.1158/2767-9764.CRC-24-0243

**Published:** 2024-09-20

**Authors:** Ratna R. Thangudu, Michael Holck, Deepak Singhal, Alexander Pilozzi, Nathan Edwards, Paul A. Rudnick, Marcin J. Domagalski, Padmini Chilappagari, Lei Ma, Yi Xin, Toan Le, Kristen Nyce, Rekha Chaudhary, Karen A. Ketchum, Aaron Maurais, Brian Connolly, Michael Riffle, Matthew C. Chambers, Brendan MacLean, Michael J. MacCoss, Peter B. McGarvey, Anand Basu, John Otridge, Esmeralda Casas-Silva, Sudha Venkatachari, Henry Rodriguez, Xu Zhang

**Affiliations:** 1 ICF, Rockville, Maryland.; 2 Georgetown University, Washington, District of Columbia.; 3 Spectragen Informatics LLC, Bainbridge Island, Washington.; 4 University of Washington, Seattle, Washington.; 5 Leidos Biomedical, Inc., Rockville, Maryland.; 6 Center for Biomedical Informatics & Information Technology, National Cancer Institute, Rockville, Maryland.; 7 Office of Cancer Clinical Proteomics Research, National Cancer Institute, Rockville, Maryland.

## Abstract

Proteomics has emerged as a powerful tool for studying cancer biology, developing diagnostics, and therapies. With the continuous improvement and widespread availability of high-throughput proteomic technologies, the generation of large-scale proteomic data has become more common in cancer research, and there is a growing need for resources that support the sharing and integration of multi-omics datasets. Such datasets require extensive metadata including clinical, biospecimen, and experimental and workflow annotations that are crucial for data interpretation and reanalysis. The need to integrate, analyze, and share these data has led to the development of NCI’s Proteomic Data Commons (PDC), accessible at https://pdc.cancer.gov. As a specialized repository within the NCI Cancer Research Data Commons (CRDC), PDC enables researchers to locate and analyze proteomic data from various cancer types and connect with genomic and imaging data available for the same samples in other CRDC nodes. Presently, PDC houses annotated data from more than 160 datasets across 19 cancer types, generated by several large-scale cancer research programs with cohort sizes exceeding 100 samples (tumor and associated normal when available). In this article, we review the current state of PDC in cancer research, discuss the opportunities and challenges associated with data sharing in proteomics, and propose future directions for the resource.

**Significance:**

The Proteomic Data Commons (PDC) plays a crucial role in advancing cancer research by providing a centralized repository of high-quality cancer proteomic data, enriched with extensive clinical annotations. By integrating and cross-referencing with complementary genomic and imaging data, the PDC facilitates multi-omics analyses, driving comprehensive insights, and accelerating discoveries across various cancer types.

## Introduction

**Figure 1:**
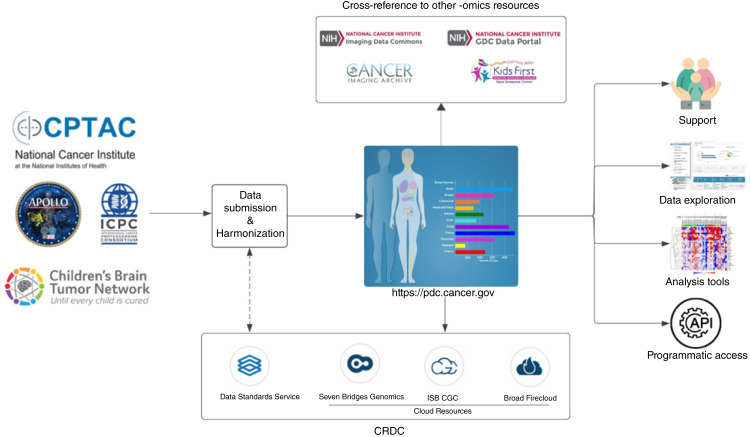
Overview of NCI’s PDC: harmonized data distribution and interoperability within NCI’s CRDC.

Precision medicine aims to tailor medical treatment to the unique characteristics of individual patients, with the goal of improving outcomes and reducing healthcare costs. One key challenge in precision medicine is the need for large, high-quality datasets that can be used to identify biomarkers and other molecular features associated with disease susceptibility, progression, and response to treatment. Toward this goal, large consortiums, such as The Cancer Genome Atlas (ref. 1) and the Clinical Proteomic Tumor Analysis Consortium (CPTAC; ref. 2), have generated vast amounts of multi-omics data from thousands of patient samples across multiple cancer types. Historically, several proteomic repositories, such as PRIDE (3), PeptideAtlas (4), and MassIVE (University of California San Diego, USA, in 2014), under the umbrella of ProteomeXchange (5), an international consortium, have supported the data sharing in proteomics. However, there is a growing need for resources that support the sharing and integration of multi-omics datasets with extensive clinical annotations, with emphasis on data reuse. This necessitates a resource that encompasses data from thousands of patient samples across various national and international programs, covering multiple cancer types and stages. The resource should (1) facilitate integration of proteomic, genomic, and other omics data to advance the discovery of new biomarkers and therapeutic targets, and (2) provide comprehensive annotation with clinical metadata, allowing researchers to delve into the connections between molecular features and clinical outcomes. With this objective in mind, the NCI has established Cancer Research Data Commons (CRDC; refs. 6, 7), a cloud-based data science infrastructure that provides secure access to a large, comprehensive, and expanding collection of cancer research data and analytical tools. Proteomic Data Commons (PDC) is a key component of this effort, providing a central repository for proteomic data that can be easily accessed and analyzed by researchers across the cancer research community (Fig. 1). Along with other data nodes such as the Genomic Data Commons (GDC; ref. 8) and Imaging Data Commons (IDC; ref. 9), the PDC provides a comprehensive repertoire of cancer proteomic data (10). By integrating the PDC resource with other data commons efforts, we aim to accelerate the pace of discovery in cancer research and improve the lives of patients with cancer.

In this article, we describe the design and implementation of the PDC, highlight its key features, and describe how it interoperates with other resources within the NCI’s cancer ecosystem, the CRDC.

## Materials and Methods

### Data model and data dictionaries

We have developed a robust data model and data dictionary to ensure effective organization and standardized representation of the proteomic data. The PDC data model serves as a structured framework for capturing and organizing diverse types of proteomic information, including experimental metadata, biospecimen and clinical information, raw data, and analytical results. The data are represented as various entities such as administrative (program and project), biospecimen hierarchy (case, sample, and aliquot), clinical (demographic, diagnosis, follow-up, treatment, exposure, and family history), experimental design, protocol, and file metadata. It ensures consistency and facilitates data harmonization across different datasets, allowing researchers to easily compare and analyze proteomic data from multiple sources. The data model outlines the complex sample to data file relationships resulting from sample multiplexing and fractionation that are common in proteomic experiments.

The accompanying data dictionary provides a comprehensive guide to the definitions, formats, and conventions used within the PDC data model for each of the data elements for the entities. It serves as a reference for data contributors and users, ensuring clear and standardized data representation. The data dictionaries are based on community standards, ontologies, and controlled vocabularies, such as those from cancer Data Standards Registry and Repository (ref. 11), International Classification of Diseases (ref. 12), and HUPO Proteomics Standards Initiative mass spectrometry (PSI-MS; ref. 13). We also align with the CRDC Data Standards Service, which aims to define standard data elements across all CRDC Data Commons (14).

### Design and development

PDC has been built on Amazon Web Services cloud platform to take advantage of its benefits in terms of scalability, security, accessibility, and collaborative research. The infrastructure is Federal Information Security Management Act compliant with stringent access controls, encryption protocols, regular audits, and incident response procedures, guaranteeing the confidentiality, integrity, and availability of data from potential threats and vulnerabilities.

During the initial design phase in 2017 to 2018, a minimum viable product was created with two key components to support data submission and data distribution. A variety of proteomic data publicly available from the CPTAC program was populated during the design phase to serve as testing data. The minimum viable product resource was made available to the public, and the feedback collected was used to improve the system during the actual build phase, which was officially launched in March 2020.

### Data harmonization

By adhering to the principles of Findability, Accessibility, Interoperability, and Reusability (FAIR) of data (ref. 15), the PDC harmonizes proteomic data from various programs such as the CPTAC, Children’s Brain Tumor Network, International Cancer Proteogenome Consortium (ICPC), and Applied Proteogenomics OrganizationaL Learning and Outcomes (APOLLO; ref. 16), using standardized workflows and community-accepted ontologies. This process effectively eliminates the “data pipeline” variable, thus facilitating comparisons across datasets.

The first step in the harmonization process assigns standard identifiers; performs data integrity checks; and ensures adherence to standards (community-accepted vocabulary and nomenclature for clinical attributes, peptides, proteins, protein sequence variants, and modifications and open data formats for files) and the PDC data model. The second step involves organizing the data to prevent duplication. Given that most cancer cohorts in PDC undergo multiple characterizations [such as global proteomics, posttranslational modifications (PTMs), metabolomics, and lipidomics], the data are meticulously organized to represent cases or subjects and their associated clinical data without duplication across these different data types. This approach not only prevents data redundancy but also simplifies data management and retrieval, enhancing the efficiency of research and enabling comprehensive multi-omics analyses.

The final step in the harmonization is to process the submitted raw MS data files through a Common Data Analysis Pipeline (CDAP) to produce derived analysis results, which can be used to study the identification of proteins and PTMs. Currently, CDAP harmonization is limited only to the proteomic data.

PDC primarily receives data acquired through data-dependent acquisition (DDA; ref. 17) and data-independent acquisition (DIA; ref. 18) MS methods and employs different CDAPs to process them. Following analysis by either CDAP, the results—mzML spectral data files, Peptide Spectral Match (PSM) results, and summary reports for proteins and PTM sites—are made available on the PDC Data Portal, along with the original raw data and metadata.

### Common data analysis pipeline

The CDAP was initially developed to encourage the reuse of the data acquired through DDA methods from the CPTAC consortium by the research community (19). The DDA CDAP supports a wide range of experimental designs analyzing patient-derived samples and human-in-mouse xenografts for whole proteome, enriched phosphopeptides, aceytlpeptides, deglycosylated N-glycopeptides, and ubiquitilated peptides, using isotopic labels, to create analytical samples that multiplex biological samples along with a study-wide common internal reference sample. Since its original implementation, several enhancements to the DDA CDAP were implemented, which improved quality metrics reporting, expanded isotopic label support (TMT-10, TMT-11, TMTPro-16, and TMTPro-18), introduced additional PTM analyses (deglycosylated N-glycosite, ubiquitination site, and acetylation site), and transitioned to GENCODE 42 as the protein search database.

In addition to the DDA CDAP, a DIA CDAP is being developed that analyzes DIA MS data and harmonizes the identified peptides and proteins similar to the DDA CDAP, using the tools msconvert (20), EncyclopeDIA (21), and Skyline (22). The raw data files will be converted to mzML; the quantitative output data matrices, quality control reports, and Skyline documents will be made available through PDC. EncyclopeDIA and Skyline are open-source tools, known for their quantitative precision and accuracy. We are also exploring the use of DIA-NN (23) as an alternative to EncyclopeDIA, which will improve our support for DIA Parallel Accumulation-Serial Fragmentation on the Bruker timsTOF platform.

### Data availability

Data sharing is not applicable to this article as no data were created or analyzed in this study.

## Results

Currently, the PDC primarily hosts MS-based proteomic data generated from large consortia such as the CPTAC, ICPC, and APOLLO. Studies include proteome, phosphoproteome, glycoproteome, acetylome, and ubiquitylome data obtained from DDA or DIA approaches either by label-free or isobaric-labeling workflows using isobaric tag for relative and absolute quantitation (iTRAQ) or tandem mass tag (TMT) reagents. More recently, PDC started distributing MS-based metabolomic and lipidomic data alongside the complementary proteomic data. The data are linked to accompanying genomic and imaging data within CRDC, if available. Since launch, PDC has released more than 160 datasets derived from nearly 4,000 patient samples from 19 cancer types. All data in PDC are open access, and the website sees an average of 5,000 users per month from around the world.

The CPTAC Public Data Portal (24), crucial for disseminating proteomic data from the CPTAC consortium, was retired in 2021, and all of the data were migrated to PDC, making it compliant with FAIR principles, ensuring accessibility, and facilitating integration with other multi-omics datasets within the CRDC.

### Data portal

PDC home page summarizes the available data across the resource by disease type and primary site of cancer. The *Explore* page provides an interface for exploring available data, defining cohorts of cases, and accessing and analyzing data. By utilizing the highly curated clinical, biospecimen, and experimental metadata, the Explore page facilitates a faceted search whereby users can identify the studies of their interest. Furthermore, users can search by various identifiers including case, sample, aliquot, study, gene, and protein accessions.

**Figure 2:**
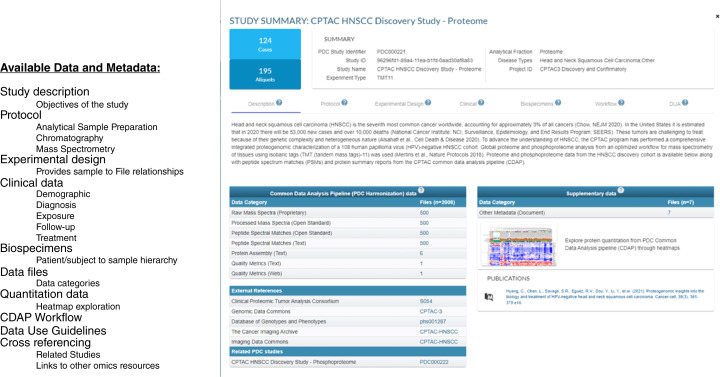
PDC study summary page offering a comprehensive overview of both data and metadata.

In PDC, a study refers to a collection of instrument raw files generated using a specific experimental protocol. It includes a well-annotated experimental design (sample to file mapping) and can be analyzed through a single bioinformatics pipeline. Consequently, the various global proteome and PTM characterizations are each represented as unique studies. The individual study summary page provides a comprehensive overview through a study description, the details of the sample preparation, chromatography and MS protocols used, biospecimen and clinical information, experimental design that provides sample to data file relationships, workflow metadata providing the parameters used in the CDAP, available data organized by file categories, publications, links to other resource where complementary genomic, imaging data for the same cohort may exist (Fig. 2). The case (subject/participant/donor) summary pages provide comprehensive information on the biospecimen hierarchy of the samples and aliquots derived from the subject, their clinical data, and a list of studies they have been part of. Reuse of complex multi-omics data from large proteogenomic consortiums is often hindered by the challenges in accurately identifying the complementary omics data available across multiple resources. PDC addresses this by providing extensive references to external resources such as GDC, The Cancer Imaging Archive (25), Database of Genotypes and Phenotypes (26), and Sequence Read Archive (27). These references are available both at the case level and at the study level wherever possible, which can help alleviate the difficulties associated with integrating multi-omics data.

The available data types in PDC (Table 1) include raw MS files in proprietary (vendor) format as submitted by the authors and several outputs from the CDAP processing which include raw data transformed to HUPO PSI mzML format, PSM information in tab separated and HUPO PSI mzIdentML format, protein summary reports for proteins and PTM sites and quality metrics files reporting the statistics of the MS/MS spectra of the datafiles. Quantitative information in the PSM and protein reports contain the spectrum-level or gene-level (“rolled-up”) precursor peak areas and spectral counts for label-free or reporter ion log-ratios for labeled multiplexing experiments.

**Table 1:** Available data types in the proteomic data commons

Data Type	Description
Raw MS data	‐ Raw (vendor) format: MS data uploaded by the data submitters as RAW or vendor format files corresponding to the mass spectrometers used to acquire the spectra
	‐ mzML format: RAW format spectra in the HUPO PSI compliant mzML format, generated by PDC
Peptide-spectrum match (PSM) Data	Generated by PDC CDAP
	‐ RAW PSM format: The best PSMs, from the first-level analysis of the PDC CDAP, for each tandem-mass spectrum against the peptide sequences from a reference protein sequence database (UniProt) in tsv format
	‐ mzIdentML PSM format: Raw PSMs in the PSI compliant mzIdentML format
Protein assembly	Generated by PDC CDAP
	*DDA analysis:*
	**.summary.tsv**—protein identification summary report
	**.precursor_area.tsv**—label-free workflow protein quantitation report for relative quantitation by precursor peak area integration
	**.spectral_count.tsv**—label-free workflow protein quantitation report for relative quantitation by spectral counts
	**.itraq.tsv**—iTRAQ workflow protein relative quantitation report
	**.tmt.tsv**—TMT workflow protein relative quantitation report
	**.peptides.tsv**—identified peptide summary report
	**.phosphopeptide.tsv**—labeled workflow phosphopeptide relative quantitation report
	**.phosphosite.tsv**—labeled workflow phosphopeptide relative quantitation report
	**.glycopeptide.tsv**—labeled workflow N-linked glycopeptide relative quantitation report
	**.glycosite.tsv**—labeled workflow N-linked glycosite relative quantitation report
	*DIA analysis:*
	**precursors_unnormalized.tsv**—unnormalized precursor peak areas
	**precursors_normalized.tsv**—median normalized precursor peak areas
	**proteins_unnormalized.tsv**—unnormalized protein abundances. Calculated by taking the sum of every precursor in the protein
	**proteins_normalized.tsv**—DirectLFQ normalized protein abundances
	**sky.zip**—the skyline document used for quantification of chromotographic peaks
QC reports	Quality control metrics computed by the CDAP; the report consists of summary statistics derived from all MS/MS spectra from the raw spectral data files.
Supplementary data	**Other metadata provided by data submitters, including descriptive protocols, clinical metadata, and other useful information. Data submitters may also provide processed outputs from the analysis pipelines used in their peer-reviewed publications**.

In addition to the CDAP harmonized data, PDC also encourages authors to submit the processed data discussed in their publication. These data are accessible as supplementary information on the study summary pages and through the PDC publications page. The latter catalogs articles from available studies in PDC and offers convenient access to the processed data described in those articles. This page is designed to assist researchers who often discover data through research papers.

In addition to data files, the PDC annotates the CDAP protein reports with rich clinical data and offers the ability to visualize protein quantitation data using a data analysis and matrix visualization tool (https://software.broadinstitute.org/morpheus) for exploring relative quantitation data as heatmaps. Users can cluster data based on the clinical annotations, generate new annotations, and interact with features such as searching, filtering, and sorting using gene names and sample identifiers, along with displaying charts and other functionalities. Individual PDC study summary pages provide links to the heatmaps when available. All the heatmaps are also available through dedicated analysis page for quantitative data exploration for easy access.

PDC data are also accessible for in-depth analysis through specialized tools like PepQuery (28), a universal targeted peptide search engine. It is designed to identify or validate known and novel peptides of interest in both local and publicly available MS-based proteomic datasets. Additionally, cProsite (29) offers a web-based interactive platform for online analysis of proteomic, phosphoproteomic, and genomic data.

### Application programming interface

PDC offers GraphQL-based Application Programming Interfaces (API), which provides more flexibility than REST APIs to request specific data, reducing over-fetching and enabling more efficient data fetching. Users can find swagger-based documentation, a playground to try the APIs and sample Python notebooks to provide guidance on running API queries, visualizing data, and performing statistical analyses (Table 2).

**Table 2:** Documentation resources in the PDC

Category	Location	Overview
Data model and dictionaries	https://pdc.cancer.gov/pdc/data-dictionary	Comprehensive guide to PDC’s data model and dictionaries
Data submission tutorial	https://pdc.cancer.gov/pdc/submit-data	Step-by-step instructions for data submission
CDAPs	https://pdc.cancer.gov/pdc/harmonization	Overview of PDC’s CDAPs
API documentation	https://pdc.cancer.gov/pdc/api-documentation	Documentation and examples for PDC’s APIs
Frequently asked questions	https://pdc.cancer.gov/pdc/faq	FAQ section addressing common user queries
Data submission and sharing policies	https://pdc.cancer.gov/pdc/data-use-guidelines	Guidelines and policies for data usage and sharing
PDC CPTAC pan-cancer resource	https://pdc.cancer.gov/pdc/cptac-pancancer	Information and datasets related to CPTAC pan-cancer studies
Analyze PDC data in the cloud	https://pdc.cancer.gov/pdc/cloud-data-analysis	Techniques for analyzing PDC data in NCI CRDC cloud infrastructure
Analyze PDC quantitation data	https://pdc.cancer.gov/pdc/explore-quantitation-data	Analyzing and interpreting quantitation data from PDC

### Study identifiers and versioning

PDC ensures data reliability and traceability through persistent identifiers and versioning. Persistent identifiers help in citing data, whereas versioning tracks changes over time, enabling researchers to access specific dataset versions for reproducible analyses. Older versions remain accessible if changes are related to advancements in analysis methods or if their availability is deemed necessary for data reuse.

### Multi-omics integration and data analysis in the cloud

**Figure 3:**
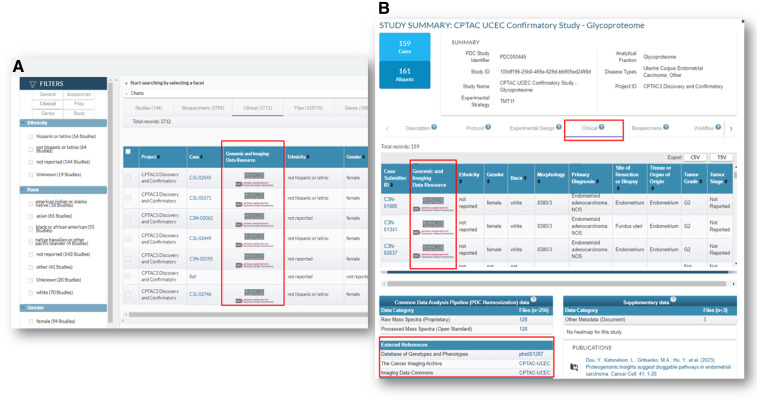
Cross referencing to genomic and imaging resources for individual cases. Example of cross-referencing (**A**) on the Clinical tab of PDC’s Explore page; **B,** on the Clinical tab and External References section of PDC study summary pages.

PDC and CRDC aim to simplify data reuse by facilitating access to complex datasets and analysis tools. Programs like CPTAC and APOLLO distribute multi-omics data across CRDC nodes—PDC for proteomic data, GDC for genomic data, and IDC for imaging data. PDC displays the cross-referencing to these resources for individual cases on portal, allowing users to download information via manifest files and APIs (Fig. 3). Whereas PDC offers curated data, NCI Cloud Resources (CR) provide cloud-based computational infrastructure and analysis tools (30). PDC enables interoperability with CRs, allowing users to identify and transfer data seamlessly for analysis on platforms like Seven Bridges Genomics Cancer Genomics Cloud (31) or Broad Institute’s Firecloud (bioRxiv 209494). Additionally, PDC APIs integrate with ISB-CGC (32), providing access to quantitative data alongside genomic data on the Google BigQuery infrastructure. This integration empowers users to perform multi-omics analyses by combining protein quantitation with complementary genomic data within the CRDC ecosystem. More details on this integration and usage can be found on the PDC portal.

### CPTAC pan-cancer resource

To facilitate pan-cancer investigations, CPTAC researchers have created a vast proteogenomic resource covering global proteomics, PTMs, genomics, miRNA, total RNA sequencing, DNA methylation, imaging, and clinical information for 1,000+ patients with cancer across 10 tumor types. PDC has established a unified Pan-Cancer page to serve as one of the resources for these data (33). This page offers data dissemination, research papers, supplementary materials, and links to Cancer Data Service for protected genomic data.

### Data submission portal

The data submission portal was developed using the Chorus open-source project (https://chorusproject.org/), a MS-based proteomic resource where researchers can upload raw data, organize them by the instrument make and model, create projects, and share with other members of their program. Substantial enhancements were made to facilitate the inclusion of various metadata including the protocols, clinical and biospecimen, experimental design, and file attributes in adherence with the PDC data model and data dictionaries. The data submission portal serves as the workspace for data submitters, especially those who do multiple data submissions over time, to organize the studies into programs and projects. We used the open-source concept to save development time and promote the reuse of established resources.

PDC provides extensive documentation of the resource; its data model, its various features, APIs, and the bioinformatics pipelines used to harmonize the data are provided in Table 2.

### Data submission and sharing policies

PDC hosts cancer-related proteomic data collected primarily from human subjects through MS-based approaches, supported by extensive data and metadata annotations. All data are freely accessible to the public under Creative Commons (CC-BY) license policy. Users can request to deposit their data through a data submission request process. PDC carefully evaluates potential submission requests based on the clarity of the biological question, data quality and quantity, potential impact on cancer proteomics, and Institutional Review Board approvals to ensure ethical standards are met. Additionally, PDC accepts only de-identified data and verifies that submissions do not include personally identifiable information.

PDC supports NIH data sharing policy, emphasizing prompt accessibility of shared scientific data, ideally concurrent with an associated publication or by the end of the award/support period, whichever comes first.

## Discussion

Over the past decade, data sharing has transformed from a recommended practice to a mandate by many scientific journals and funding organizations to ensure the accessibility of high-value datasets to aid in accelerating the pace of biomedical research. This has led to significant volumes of MS-based proteomic data being freely available in the public domain (5). However, the effective use, reuse, reprocessing, and repurposing of this complex data for new discoveries, especially in cancer research, are contingent upon the presence of highly curated and standardized metadata and clinical annotations. The PDC has been established to address this gap. In this article, we described a comprehensive data resource that distributes MS-based cancer-related proteomic data from large cancer proteogenomic programs and facilitates proteogenomic integration through interoperability with other data commons and analytical resources within the NCI CRDC. The resource serves both as a data repository and as a knowledge base. The extensive curation of the biospecimen, clinical, and proteomic metadata provides an intuitive cohort exploration using biospecimen, clinical, gene, data, and proteomic attributes and facilitates combing data from diverse sources for meta-analysis. We have recently expanded the PDC data model and dictionaries to incorporate MS-based metabolomic and lipidomic data alongside the complementary proteomic data. Despite metabolomics and lipidomics being distinct omics types, the PDC data model has been adjusted to accommodate these differences. This update now allows PDC to host comprehensive MS-based multi-omics datasets generated for the same cohorts from the CPTAC consortium, enhancing the integration and utility of diverse data types.

To promote the reuse of data, we designed the data model and dictionaries to align with existing resources and community standards such as the cancer Data Standards Registry and Repository and HUPO PSI. This approach simplifies the CRDCs efforts to create a common data model that users can search using variables such as participant, sample, tissue, disease, or race. This aggregation also makes it possible for researchers to create complex multistudy datasets from both open- and controlled-access datasets across the CRDC that can be used for integrative analysis.

Despite the robust capabilities of the PDC, several challenges remain. The extensive metadata requirements, while ensuring data reliability and reusability, impose a significant burden on both data submitters and PDC data managers. Ensuring the completeness and accuracy of data relies heavily on the submitters’ expertise, necessitating continuous updates to our data dictionaries and comprehensive training resources, which is resource-intensive for the PDC. Additionally, strict enforcement of standards can delay submissions but is crucial for maintaining data quality. The integration of multimodal data presents unique challenges because of staggered releases and varying identifiers across repositories, adding complexity to data management within the PDC.

The subject matter expertise needed to manage the continuously evolving data standards in clinical, MS, proteomics, and metabolomics domains is critical. Adapting to these evolving standards and retrospectively updating historical data to align with new standards further complicate data management and require substantial effort and specialized knowledge. Through a suite of core standards and services, the CRDC is exploring ways to streamline the submission process, improve data interoperability, and develop tools to facilitate seamless data integration (7).

The lack of a common reference sample across proteomic datasets in the PDC is another significant challenge, as it hinders the ability to directly compare protein abundance ratios across different cancer types for the same gene, even though all data are analyzed through a CDAP. This discrepancy complicates cross-study analyses and limits the ability to draw consistent, meaningful biological conclusions. The CPTAC program is working on ways to better understand and enable experiment bridging that will facilitate such comparisons and informative visualizations.

Recent advancements in artificial intelligence technologies offer great potential to achieve the National Cancer Plan’s goal of maximizing data utility for faster progress against cancer. The PDC is working with the CRDC to lay the foundation for Artificial Intelligence Data Readiness by adhering to FAIR principles and mandating submission requirements and by encouraging data submitters to ensure the accuracy, completeness, consistency, and validity of the data.

Researchers can utilize the PDC via its interactive portal at https://pdc.cancer.gov, engage through APIs, and access them through other platforms within NCI CRDC.
